# Supplementary material for: Oral Glucocorticoid Use and Long-Term Mortality in Patients with Chronic Musculoskeletal Non-Cancer Pain: A Cross-Sectional Cohort Study
Source: Diagnostics (Basel). 2023 Jul 28;13(15):2521. doi: 10.3390/diagnostics13152521 (PMC10416933; doi:10.3390/diagnostics13152521)
Supplement: Supplementary file 1 [file diagnostics-13-02521-s001.zip › diagnostics-2502271-supplementary/Supplementary Material S1.pdf]

**Supplementary Material S1. ICD-10 codes**

Rheumatoid arthritis: M05-M06.9, M08.0-M08.89

Osteoarthritis: M13-M13.9, M15-M19.079

Low back pain: G54.1, G54.3, G54.4, G57.0-G57.12, M43.2-M43.5, M43.8, M43.9, M45-M49, M49.2-M49.89, M51-M51.9, M53, M53.2-M54, M54.1-M54.18, M54.3-M54.9, M99, M99.1-M99.9

Neck pain: G54.2, M50-M50.93, M53.0, M54.0-M54.09, M54.2

Gout: M10-M10.19, M10.3-M10.9

Other musculoskeletal disorders: I27.1, L93-L93.2, M00-M03.0, M03.2, M03.6, M07-M08, M08.9-M09.0, M09.2, M09.8, M11-M12, M12.2-M12.49, M12.8-M12.9, M14-M14.89, M22-M25.879, M30-M32.9, M34-M36.8, M40-M43.19, M65-M68.8, M70-M73, M73.8, M75-M77.9, M80-M83.4, M83.8-M87.09, M87.3-M89.59, M89.7-M95.9, M99.0-M99.09
