# Supplementary material for: Oral Glucocorticoid Use and Long-Term Mortality in Patients with Chronic Musculoskeletal Non-Cancer Pain: A Cross-Sectional Cohort Study
Source: Diagnostics (Basel). 2023 Jul 28;13(15):2521. doi: 10.3390/diagnostics13152521 (PMC10416933; doi:10.3390/diagnostics13152521)
Supplement: Supplementary file 1 [file diagnostics-13-02521-s001.zip › diagnostics-2502271-supplementary/Supplementary Material S2.pdf]

Supplementary Material S2. ICD-code for disease specific mortality

The disease specific mortality includes infectious mortality (A00-B99), cancer mortality (C00-D49), blood disease mortality (D50-D89), endocrine disease mortality (E00-E89), mental disease mortality (F01-F99), nervous disease mortality (G00-G99), circulatory disease mortality (I00-I99), respiratory disease mortality (J00-J99), digestive disease mortality (K00-K95), skin disease mortality (L00-L99), musculoskeletal disease mortality (M00-M99), genitourinary disease mortality (N00-N99), mortality due to event during pregnancy, childbirth and the puerperium (O00-O9A), congenital disease mortality (Q00-Q99), mortality associated symptoms, signs and abnormal clinical and laboratory findings (R00-R99), mortality due to Injury, poisoning and certain other consequences of external causes (S00-T88), and mortality due to factors influencing health status and contact with health services (Z00-Z99).
