# Supplementary material for: Oral Glucocorticoid Use and Long-Term Mortality in Patients with Chronic Musculoskeletal Non-Cancer Pain: A Cross-Sectional Cohort Study
Source: Diagnostics (Basel). 2023 Jul 28;13(15):2521. doi: 10.3390/diagnostics13152521 (PMC10416933; doi:10.3390/diagnostics13152521)
Supplement: Supplementary file 1 [file diagnostics-13-02521-s001.zip › diagnostics-2502271-supplementary/Supplementary Material S4.pdf]

Supplementary Material S4. GC exposure during 2011-2019

	GC use in 2011	GC use in 2012	GC use in 2013	GC use in 2014	GC use in 2015	GC use in 2016	GC use in 2017	GC use in 2018	GC use in 2019
GC user in 2010 n=9,038	5,208 (57.6)	3,969 (43.9)	3,358 (37.2)	2,918 (32.3)	2,587 (28.6)	2,353 (26.0)	2,138 (23.7)	1,932 (21.4)	1,735 (19.2)
Non-GC users in 2010 n=1,794,981	4,485 (0.2)	6,359 (0.4)	7,298 (0.4)	8,084 (0.5)	8,991 (0.5)	9,492 (0.5)	10,159 (0.6)	10,996 (0.6)	11,603 (0.6)

GC, glucocorticoid
