# Supplementary material for: Oral Glucocorticoid Use and Long-Term Mortality in Patients with Chronic Musculoskeletal Non-Cancer Pain: A Cross-Sectional Cohort Study
Source: Diagnostics (Basel). 2023 Jul 28;13(15):2521. doi: 10.3390/diagnostics13152521 (PMC10416933; doi:10.3390/diagnostics13152521)
Supplement: Supplementary file 1 [file diagnostics-13-02521-s001.zip › diagnostics-2502271-supplementary/Supplementary Material S5.pdf]

Supplementary Material S5. Multivariable time-dependent Cox regression model for all-cause mortality during 2012-2019 among cohort with CNCP in 2010.

Variable	HR (95% CI)	*P*-value
GC user (vs control)	1.45 (1.36, 1.54)	<0.001

CNCP, chronic non-cancer pain; HR, hazard ratio; CI, confidence interval; GC, glucocorticoid
